# Explainable artificial intelligence for microbiome data analysis in colorectal cancer biomarker identification

**DOI:** 10.3389/fmicb.2024.1348974

**Published:** 2024-02-15

**Authors:** Pierfrancesco Novielli, Donato Romano, Michele Magarelli, Pierpaolo Di Bitonto, Domenico Diacono, Annalisa Chiatante, Giuseppe Lopalco, Daniele Sabella, Vincenzo Venerito, Pasquale Filannino, Roberto Bellotti, Maria De Angelis, Florenzo Iannone, Sabina Tangaro

**Affiliations:** ^1^Dipartimento di Scienze del Suolo, della Pianta e degli Alimenti, Università degli Studi di Bari Aldo Moro, Bari, Italy; ^2^Istituto Nazionale di Fisica Nucleare, Sezione di Bari, Bari, Italy; ^3^Dipartimento di Medicina di Precisione e Rigenerativa e Area Jonica, Università degli Studi di Bari Aldo Moro, Bari, Italy; ^4^Dipartimento Interateneo di Fisica M. Merlin, Università degli Studi di Bari Aldo Moro, Bari, Italy

**Keywords:** machine learning, explainable artificial intelligence, colorectal cancer, microbiome, biomarker identification, microbiota, precision medicine

## Abstract

**Background:**

Colorectal cancer (CRC) is a type of tumor caused by the uncontrolled growth of cells in the mucosa lining the last part of the intestine. Emerging evidence underscores an association between CRC and gut microbiome dysbiosis. The high mortality rate of this cancer has made it necessary to develop new early diagnostic methods. Machine learning (ML) techniques can represent a solution to evaluate the interaction between intestinal microbiota and host physiology. Through explained artificial intelligence (XAI) it is possible to evaluate the individual contributions of microbial taxonomic markers for each subject. Our work also implements the Shapley Method Additive Explanations (SHAP) algorithm to identify for each subject which parameters are important in the context of CRC.

**Results:**

The proposed study aimed to implement an explainable artificial intelligence framework using both gut microbiota data and demographic information from subjects to classify a cohort of control subjects from those with CRC. Our analysis revealed an association between gut microbiota and this disease. We compared three machine learning algorithms, and the Random Forest (RF) algorithm emerged as the best classifier, with a precision of 0.729 ± 0.038 and an area under the Precision-Recall curve of 0.668 ± 0.016. Additionally, SHAP analysis highlighted the most crucial variables in the model's decision-making, facilitating the identification of specific bacteria linked to CRC. Our results confirmed the role of certain bacteria, such as *Fusobacterium, Peptostreptococcus*, and *Parvimonas*, whose abundance appears notably associated with the disease, as well as bacteria whose presence is linked to a non-diseased state.

**Discussion:**

These findings emphasizes the potential of leveraging gut microbiota data within an explainable AI framework for CRC classification. The significant association observed aligns with existing knowledge. The precision exhibited by the RF algorithm reinforces its suitability for such classification tasks. The SHAP analysis not only enhanced interpretability but identified specific bacteria crucial in CRC determination. This approach opens avenues for targeted interventions based on microbial signatures. Further exploration is warranted to deepen our understanding of the intricate interplay between microbiota and health, providing insights for refined diagnostic and therapeutic strategies.

## 1 Introduction

Colorectal cancer (CRC) stands as the third most prevalent cancer globally (Morgan et al., 2023), claiming a significant toll in cancer-related fatalities. The high mortality is due to the abnormal growth of cells with the capacity to invade tissues and spread to other parts of the body. Most colorectal cancers are due to lifestyle and advanced age and only a few cases are attributable to hereditary genetic diseases. Its incidence is constantly increasing, and in-depth understanding of the pathogenetic mechanisms, early diagnosis and innovative therapeutic options have become crucial imperatives to address this growing challenge. The complexity of colorectal cancer is highlighted by the diversity of pathological pathways involved and the variability in response to treatments. The prevailing gold standard for CRC diagnosis, colonoscopy, is burdened by invasiveness and discomfort. However, resistance to conventional treatments, post-surgical recurrence and the need to improve access to care, especially in disadvantaged communities make it necessary to open up to personalized therapies and more targeted management strategies. A non-standardized approach keep in mind the peculiar molecular characteristics of each tumor and the patient's individual responses to therapies. Hence, the pressing demand for non-invasive, cost-effective early detection methods persists. Non-invasive therapies take on particular relevance with a view to reducing physical and psychological stress on patients, reducing the recovery period and improving the quality of life post-treatment.

The gut microbiota, a complex community of microorganisms that colonize the gastrointestinal tract, has emerged as a critical player in the regulation of intestinal homeostasis and the modulation of local immune responses. In recent years, a growing body of scientific evidence has highlighted the critical role of the intestinal microbiota in the pathogenesis and development of colorectal cancer. The dynamic interactions between the microbiota and the intestinal mucosa play a key role in maintaining a physiological environment and preventing the onset of cellular alterations. However, dysbiosis or imbalances in the composition of the microbiota can contribute to carcinogenesis, promoting chronic inflammation, the production of carcinogenic metabolites and alteration of the mucosal barrier. Certain bacteria, like *Fusobacterium nucleatum* and *Parvimonas micra*, are notably more abundant in CRC patients, often linked to the disease's development (Yachida et al., 2019; Löwenmark et al., 2020; Wu et al., 2021). These findings drive the exploration of using fecal biomarkers for CRC diagnosis. Understanding the central role of the gut microbiota in the context of colorectal cancer could guide the development of personalized strategies for disease management, exploiting the therapeutic potential of microbial manipulation. Harnessing the power of machine learning (ML) (Amodeo et al., 2021; Bellando-Randone et al., 2021; Rynazal et al., 2023; Golob et al., 2024), our study crafts a comprehensive framework to scrutinize fecal microbiome data gleaned from both healthy subjects and those afflicted with CRC. This framework intricately involves data preprocessing, feature extraction, feature selection, and model construction, employing an array of ML algorithms. To ensure transparency and interpretability in our study, we embrace the principles of Explainable Artificial Intelligence (XAI) (Lombardi et al., 2021a,b; Bellantuono et al., 2023; Novielli et al., 2023). XAI not only enhances the trustworthiness of our models but also empowers clinicians to understand the rationale behind each prediction. This is particularly crucial in the context of personalized CRC management, where treatment decisions need to be aligned with the unique characteristics of each patient. The impact of gut microbiota on CRC analyzed through machine learning, coupled with transparent explanations afforded by XAI, holds the potential to develop how to diagnose and manage colorectal cancer, fostering a new era of precision medicine that is both effective and readily comprehensible.

## 2 Materials

In this study, we used three different dataset of three different works (Zackular et al., 2014; Zeller et al., 2014; Baxter et al., 2016). For each of them, we considered the control patient (NC) and the CRC ones. These datasets collect 442 human stool samples characterized by 16S metagenomic sequencing of the V4 region of the 16S rRNA, from different countries: Canada (CA), France (FRA), United States of America (USA). These dataset provide information regarding the abundance of the gut microbiota in NC patients and CRC ones at genus level. Moreover, each of them is characterized with four metadata features: gender, age, body mass index (BMI), country, as reported in Table 1.

**Table 1:** Summary table of the datasets used in the analysis.

**Dataset**	**Control**	**CRC**	**Metadata**
Baxter et al. (2016)	171	120	Gender, age, BMI, country
Zackular et al. (2014)	30	30	Gender, age, BMI, country
Zeller et al. (2014)	50	41	Gender, age, BMI, country
TOTAL	251	191	Gender, age, BMI, country

**Figure 1:**
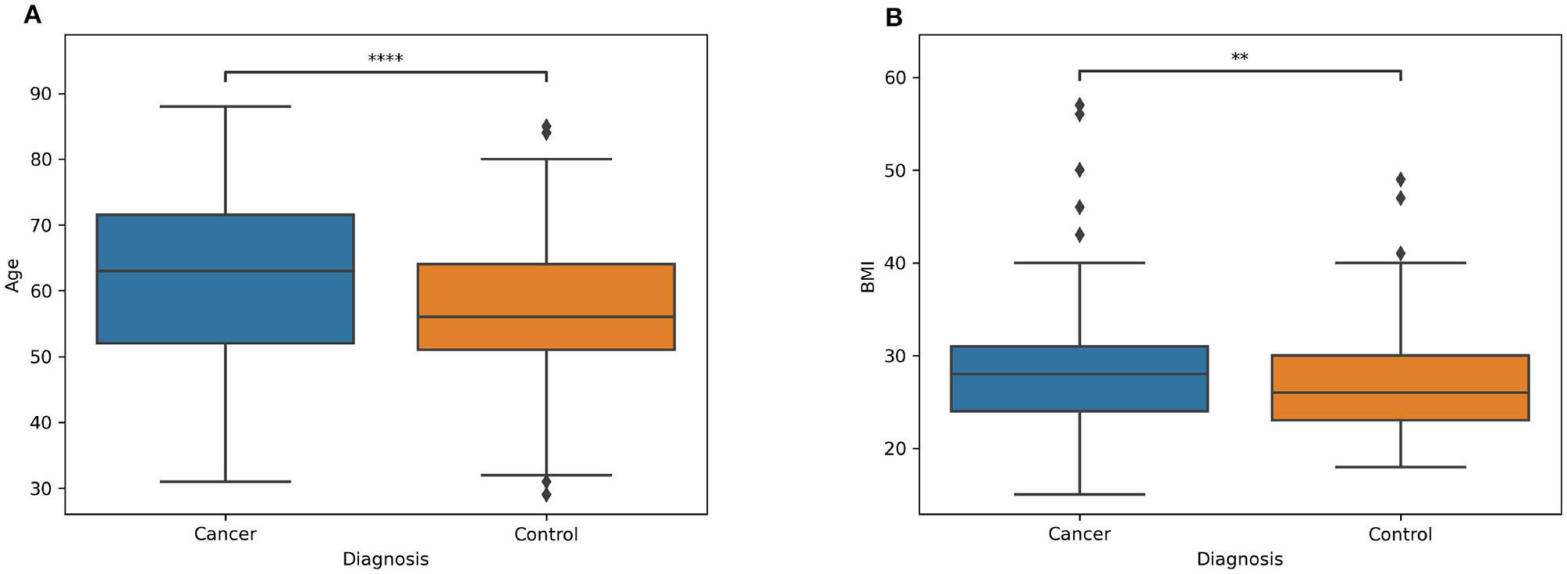
Boxplot of two classes (patients and controls) of the **(A)** age and **(B)** BMI. The symbol * denotes the significance level determined by the Mann-Whitney U rank test for comparing two distributions. **Stands for *p*-value less or equal then 0.01. ****stands for *p*-value less or equal then 0.0001.

Information about the distribution of age and BMI for both patients and controls are showed respectively in Figures 1A, B, while the demographic characteristics of the entire dataset is reported in Table 2. In the Supplementary Table S1 is reported the information related to the metadata of each subject involved in the analysis.

**Table 2:** Demographic characteristics of the study participants.

	**CRC (191)**	**Control (251)**	**p-value**
Gender	114 M / 77 F	101 M / 150 F	< 0.01
Country	2 CA / 41 FRA / 148 USA	3 CA / 50 FRA / 198 USA	0.892

The Fisher's exact test was performed for gender and country.

## 3 Methods

**Figure 2:**
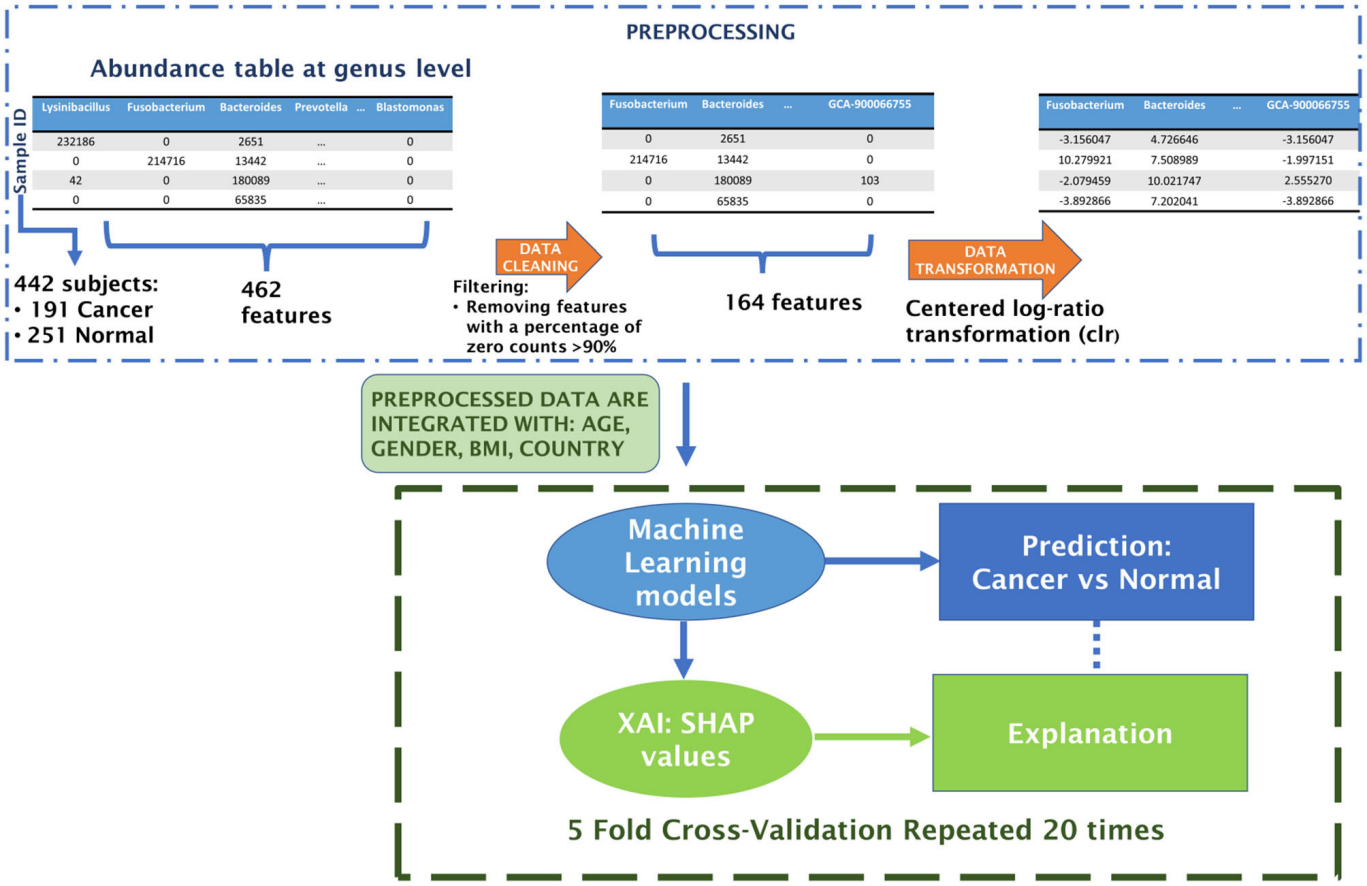
Schematic flowchart of the analysis.

The workflow begins with the preprocessing of microbiome data, followed by the construction of an explainable machine learning model. The performance of three classifiers—XGBoost, Random Forest, and Support Vector Machine—was rigorously compared through a 20-repeated 5-fold Stratified Cross Validation. Finally, we explore the functionality of the optimal classifier using the XAI approach. This includes collecting SHAP values for different (feature, prediction) pairs and averaging them across the 20 repetitions of the model CV. Figure 2 outlines the Artificial Intelligence procedure implemented in this study to develop a Machine Learning classifier for distinguishing between control and CRC samples.

### 3.1 Preprocessing of the microbiome samples

Preprocessing of microbiome data is a crucial step in the analysis pipeline (Ibrahimi et al., 2023; Papoutsoglou et al., 2023). The microbiome data undergo several preprocessing steps. Firstly, a filtration of taxonomic units is conducted, focusing on removing non-informative features or taxa that are biologically irrelevant or potential contaminants (Cao et al., 2021). This involves applying thresholds based on abundance/prevalence, variance, or correlation. In our case, low-abundance or prevalence filtering eliminates features present in <10% of the samples. The subsequent step involves normalization, aiming to address variability in sampling depth and data sparsity. One approach for data normalization is through transformation methods, wherein values are replaced with their normalized counterparts. Given that microbiome datasets are inherently compositional, these methods adhere to Aitchison's methodology for compositional data. They transform feature counts into log-ratios within each sample, utilizing an additive, centered log-ratio transformation (Aitchison, 1982; Egozcue et al., 2003).

### 3.2 Machine learning classifier

#### 3.2.1 XGBoost

The XGBoost algorithm employs a collective of decision trees trained through an iterative gradient boosting process. This process involves addressing critical points within decision trees at each step through subsequent trees. Addressing the challenge of missing values, XGBoost employs sparsity-aware split finding (Chen and Guestrin, 2016). This technique leverages data sparsity patterns in a unified manner, determining the optimal direction in the event of a missing feature necessary for a split. In the quest for optimal performance in classification under cross-validation conditions, we explore various XGBoost parameters:

- max depth ϵ {None, 3, 5},
- col sample bytree ϵ {0.1, 0.2, 0.3, 0.4, 0.5, 0.6, 0.7, 0.8, 0.9},
- n estimators ϵ {50, 100, 150, 200, 250}.

The implementation of the XGBoost algorithm utilizes the Python (version 3.11.5) package xgboost (version 2.0.2).

#### 3.2.2 Random forest

The Random Forest (RF) algorithm entails an ensemble of decision trees derived through resampling the training dataset with repetitions (bootstrapping) (Breiman, 2001). This process, along with the randomization of features during training, ensures low mutual correlation between RF trees. Decision trees generate independent predictions for each observation, and their collective outcomes are aggregated through either averaging (for regression) or majority voting (for classification). Noteworthy characteristics of RF algorithms include easy tunability, a minimal number of parameters, resilience against overfitting, the ability to assess feature importance during training, and an unbiased estimation of generalization error. In this study, we aimed to optimize the control/crc classification in cross-validation mode by varying specific RF parameters, including:

- max depth {ϵ None, 3, 5},
- n estimators ϵ {50, 100, 150, 200, 250}.

The RF algorithm implementation utilized the Python (version 3.11.5) package scikit-learn (version 1.3.0) (Pedregosa et al., 2011).

#### 3.2.3 Support vector machine

The Support Vector Machine (SVM) operates by determining the optimal boundary between two or more classes in the data space through the minimization of a loss function known as Hinge Loss, augmented with a penalty term (Cortes and Vapnik, 1995). In this algorithm, only a limited set of input observations, termed support vectors, actively contribute to delineating the boundary between classes. The SVM algorithm iterates by treating misclassified instances as support vectors, with their contribution to the loss being proportional to their distance from the boundary. This approach ensures that the loss is influenced solely by a subset of input observations, facilitating an efficient estimation of optimal parameters. For the optimization of control/CRC classification under cross-validation conditions, we vary the following SVM parameters:

- C ϵ {1, 5, 10, 20},
- Gamma ϵ {0.001, 0.01, 1}.

The SVM algorithm is implemented using the Python (version 3.11.5) package scikit-learn (version 1.3.0) (Pedregosa et al., 2011).

### 3.3 Evaluation metrics

In the realm of classification machine learning, the selection of appropriate evaluation metrics is crucial for assessing the performance of models. These metrics provide quantitative measures of a model's ability to correctly classify instances and are essential tools for comparing and optimizing different algorithms. In order to obtain statistically robust results, a 5-fold cross-validation was applied to partition the dataset, where each fold was used as a test set while the remaining four as training ones (Schaffer, 1993). An hyperparameter tuning was conducted with a random search by using the RandomizedSearchCV function of the python library scikit-learn (Bergstra and Bengio, 2012), implemented with a nested 3-fold cross- validation to avoid bias in the estimation of test error (Varma and Simon, 2006). The entire process was repeated 20 times, by dividing the dataset with different partitions between each repetition.

The metrics used to evaluate the performance of models were (Venerito et al., 2022):

- Accuracy: The accuracy is the proportion of correct predictions (both true positives and true negatives) among the total number predictions.
- Recall: The recall is a metric evaluating the frequency with which a machine learning model accurately recognizes positive instances (true positives) among all the actual positive samples. It is calculated by dividing the number of true positives by the total number of elements that actually belong to the positive class.
- Precision: The precision is a metric assessing how often a machine learning model predicts the positive class. It is computed by dividing the number of accurate positive predictions (true positives) by the total instances predicted as positive by the model (sum of true positives and false positives).
- F1 score: The F1 score is the harmonic mean of the precision and recall.
- AUC ROC: The area under the Receiver Operating Characteristic (ROC) curve;
- AUPRC: The area under the Precision-Recall (PR) curve.

We considered as positive instances those ones belonging to the CRC class.

For the evaluation of the best classifier, the one with the highest AUPRC will be chosen. This metric is well-suited for assessing the discriminative power of a classifier in the presence of an imbalanced dataset, where the number of positive cases is greater than the number of negative cases (Ozenne et al., 2015).

### 3.4 SHAP algorithm

The eXplainable Artificial Intelligence (XAI) framework encompasses a variety of techniques united by their shared focus on informativeness, uncertainty estimation, generalization, and transparency. In this study, we employ the SHAP local explanation algorithm to uncover the significance of features in classifying control/CRC samples. Serving as a local, model-agnostic *post-hoc* explainer, the SHAP algorithm derives inspiration from Shapley (SHAP) values rooted in cooperative game theory (Lundberg and Lee, 2017; Lundberg et al., 2020). It constructs interpretable linear models for individual samples, highlighting the contribution of each feature to the sample's prediction. The computation of SHAP values involves assessing the difference in model output predictions with and without specific features, considering all conceivable feature subsets. As a result, the model requires retraining on all subsets F of the complete set S of features (*F* ⊆ *S*). The SHAP value for the *jth* feature of the instance x is determined by aggregating it across all possible subsets (Equation 1):


(1) $$\begin{array}{l} \Phi \text{j}\left(\text{x}\right)=\underset{F\subseteq S-\left\{j\right\}}{\sum }\frac{|F|!\left(|S|-|F|-1\right)!}{|S|!}\left[fx\left(F\cup j\right)-fx\left(F\right)\right] \end{array}$$

where *|F |*! represents the permutations of features in the subset F, (*|S| - |F |* − 1)! the permutations of features in the subset *S -* (*F* ⊆ *{j}*) and *|S|*! is the total number of feature permutations.

The SHAP value calculation is implemented in the Python (version 3.11.5) package shap (version 0.43.0). For RF and XGBoost models, we utilized the TreeExplainer function with the “feature perturbation” parameter set to “interventional.” This approach is tailored to disrupt dependencies between features, aligning with the principles outlined in causal inference (Janzing et al., 2020). By adopting this parameter configuration, our objective was to alleviate the impact of highly correlated predictors, thereby mitigating potential misinterpretations and ensuring a more robust analysis.

## 4 Results

The objective of this study was to investigate changes in the gut microbiota among individuals with CRC in comparison to control subjects. To unveil these alterations, a machine learning-based classification model was employed, and the contribution of features was analyzed. Our attention will be directed toward the outcomes of the Artificial Intelligence workflow, specifically examining the classification performance of various Machine Learning algorithms and the prevalence of bacteroides that exerts the most significant influence on predictions.

### 4.1 Feature engineering

The dataset utilized in this study consists of abundance tables representing microbial communities from the V4 region of the 16S rRNA, collected at the genus level. Starting with an initial dataset comprising 462 features (microbial communities), the data cleaning process, as described in the methods, reduced the total number of features to 164. Following the centered log-ratio transformation for each sample, additional variables were incorporated, including country, age, BMI, and gender. This resulting dataset served as the input for the machine learning classification framework.

### 4.2 Classification CRC/control

A comprehensive correlation analysis was conducted among all features considered as inputs to the ML classifier and the output target class. The outcomes of this analysis are presented in Supplementary Figure S1, where the top features are displayed in descending order based on their correlation coefficients with the target class. Despite observing statistically significant correlations among the features, it is noteworthy that the maximum correlation does not exceed 0.3. This implies that a univariate analysis approach for classifier creation is not suitable, necessitating a multivariate approach. The limited strength of individual feature correlations underscores the need for constructing multivariate ML classification models to capture the intricate relationships within the dataset and achieve a more comprehensive understanding of the predictive factors associated with the target class.

Within this study, the efficacy of three supervised machine learning algorithms—XGB, RF, and a SVM—was assessed. The optimal classifier emerged as the one exhibiting the highest AUPRC, averaged across the 20 repetitions of the 5-fold cross-validation. As outlined in Table 3, the RF model proved to be the most proficient, excelling in terms of accuracy, precision and area under the precision-recall curve.

**Table 3:** Comparison between evaluation metrics of XGBoost (XGB), Random Forest (RF), and Support Vector Machine (SVM) classifiers.

	**ACC**	**F1**	**PREC**	**AUC ROC**	**AUPRC**
XGB	0.652 (0.017)	**0.567 (0.022)**	0.613 (0.022)	**0.701 (0.015)**	0.639 (0.021)
RF	**0.673 (0.015)**	0.507 (0.030)	**0.729 (0.038)**	0.699 (0.011)	**0.668 (0.016)**
SVM	0.633 (0.025)	0.478 (0.091)	0.613 (0.032)	0.663 (0.036)	0.597 (0.037)

The mean values accompanied by the standard deviation are shown. The highest values for each metric are indicated in bold, and the second-highest values are underscored.

**Figure 3:**
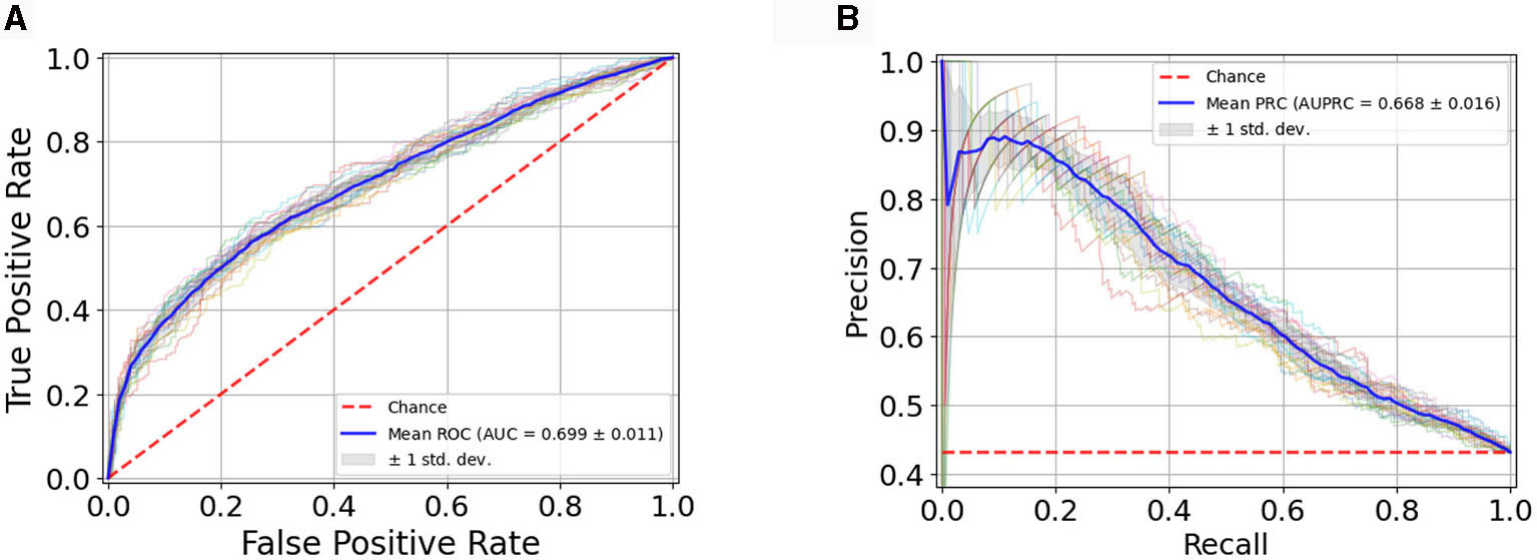
**(A)** Average ROC Curve with standard deviation over 20 model runs; **(B)** Average PR Curve with standard deviation over 20 model runs.

Figure 3 illustrates the RF classification model's performance, assessed through the Receiver Operating Characteristic (ROC) curve (Figure 3A), showcasing an Area Under the Curve (AUC) value of 0.699 ± 0.011 and through the Precision-Recall (PR) curve (Figure 3B) with an AUC of 0.668 ± 0.016. The plots showcase the average curves derived from 20 repetitions of the Cross-Validation, accompanied by their standard deviation.

In Supplementary Figures S2–S4, we present the analysis of parameter stability during the tuning phase of nested cross-validation. These figures illustrate, across multiple repetitions, the frequency with which a particular parameter was selected as the best parameter for our models. This in-depth examination provides valuable insights into the robustness and consistency of the chosen parameters throughout the nested cross-validation process.

### 4.3 Explainability

**Figure 4:**
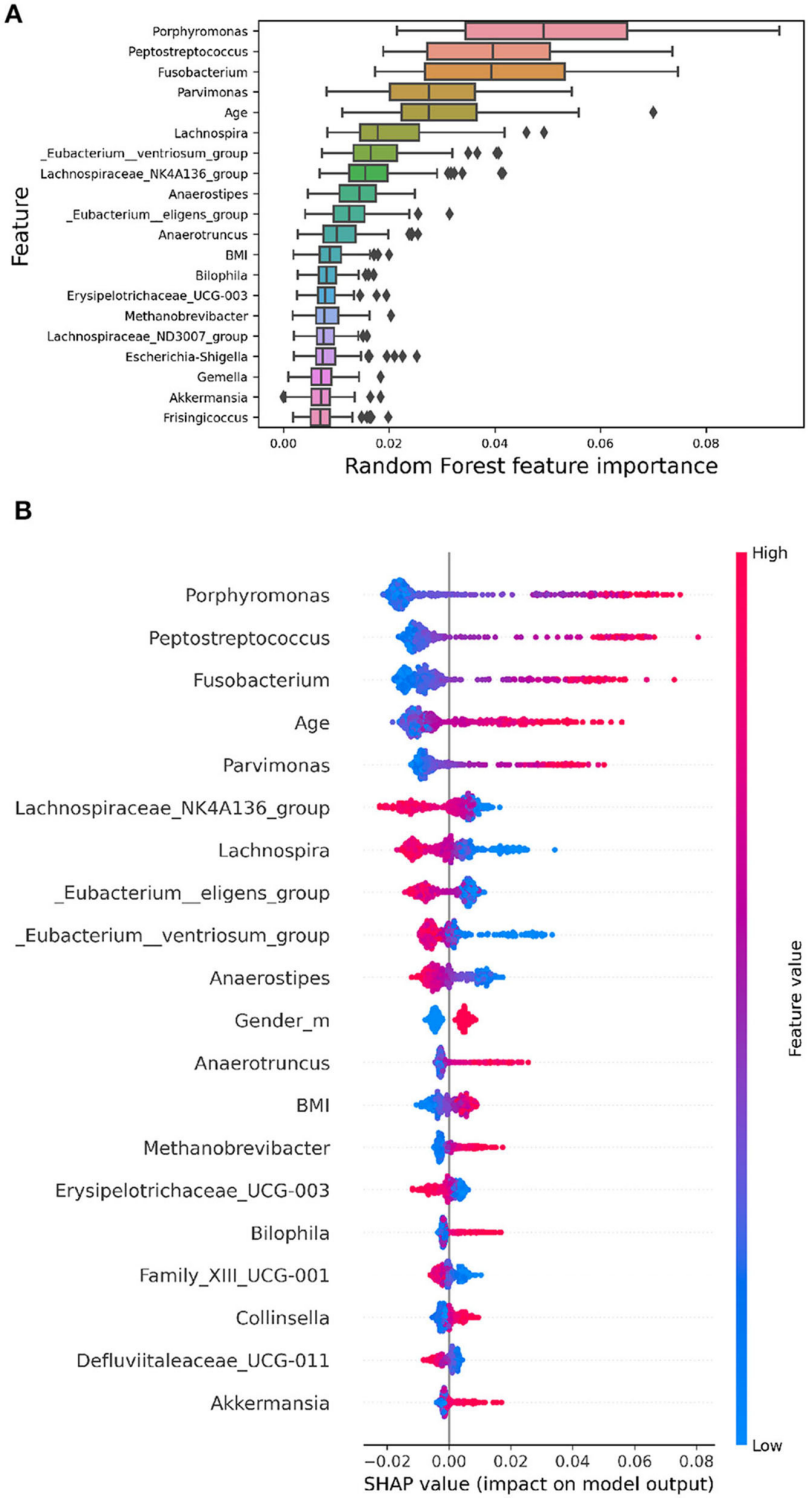
The images display the top 20 features ranked by their importance. **(A)** RF embedded feature importance. The boxplots represent the distributions of the feature importance coefficient calculated across all validation folds of the model. **(B)** SHAP summary plot depicting Shapley values for each feature. Each point represents a subject's Shapley value, with the y-axis indicating the corresponding feature and the x-axis representing the Shapley value. The color gradient reflects feature values, ranging from low to high, while features are ordered by mean importance, with more important features positioned toward the top.

Model explainability involves understanding how algorithms discern the relationship between inputs and outputs. While complex non-linear models achieve superior performance, their interpretability is often compromised. This lack of interpretability limits their application in biomedical research, where a thorough understanding of the classification process is crucial. Feature importance methods aim to quantify the contribution of each feature to the model's predictions. Global methods provide an overarching ranking of features, while local methods illuminate the contribution of each feature to a specific prediction. In Figure 4, global feature importance is illustrated using various methods.

In Figure 4A, the Random Forest embedded feature importance is presented. The importance of a feature is computed as the (normalized) total reduction of the criterion brought about by that feature, commonly referred to as the Gini importance.

Figure 4B showcases the feature importance based on SHAP values. Essentially, this method constructs an interpretable linear model around each test instance and estimates feature importance at the local level. The plot in Figure 4B reveals the most important features for classification according to the SHAP algorithm. Shapley values are calculated by averaging across all iterations of the algorithm for each subject, considering the 20 repetitions. This summary plot provides an insightful overview of each feature's relative impact on the model's predictions, contributing to a thorough understanding of the overall importance and influence of different features in the analysis.

The Figure 4B indicates the presence of bacteria, such as *Porphiromonas*, with a high relative abundance (highlighted in red points on the summary plot) on the positive side of the x-axis, while a low relative abundance (highlighted in blue points) is more prevalent on the negative side. This suggests that a higher relative abundance of these bacteria is generally associated with a higher probability value for CRC, while a lower relative abundance is linked to a lower probability value for CRC. Conversely, bacteria like *Lachnospira* exhibit the opposite pattern, implying that a high abundance of this genus is correlated with a lower probability of CRC. These nuanced insights into the direction of effects are not discernible using global explanation methods like RF's built-in feature importance. Notably, the importance rankings of features obtained from both RF and SHAP values show substantial overlap (Jaccard Index = 0.67), highlighting the robustness and stability of the model. Furthermore, the SHAP summary plot highlights that among the top 20 most significant variables, Age, Gender, and BMI are included.

We have extended our explainability analysis to include the other two models (SVM and XGBoost). Due to computational constraints, we limited the number of repetitions for SVM to 5. The SHAP summary plots for these models are now available in the Supplementary Figure S5. Additionally the Table 4 illustrates the overlap coefficient (Vijaymeena and Kavitha, 2016) between the SHAP values of the three models. Notably, we observed a higher degree of overlap between the Shapley values of the two top-performing models, RF and XGBoost.

**Table 4:** Overlap coefficient between the top 20 most important features, as determined by SHAP, across the three ML models.

RF	0.55	
XGBoost	0.40	0.75
	SVM	RF

**Figure 5:**
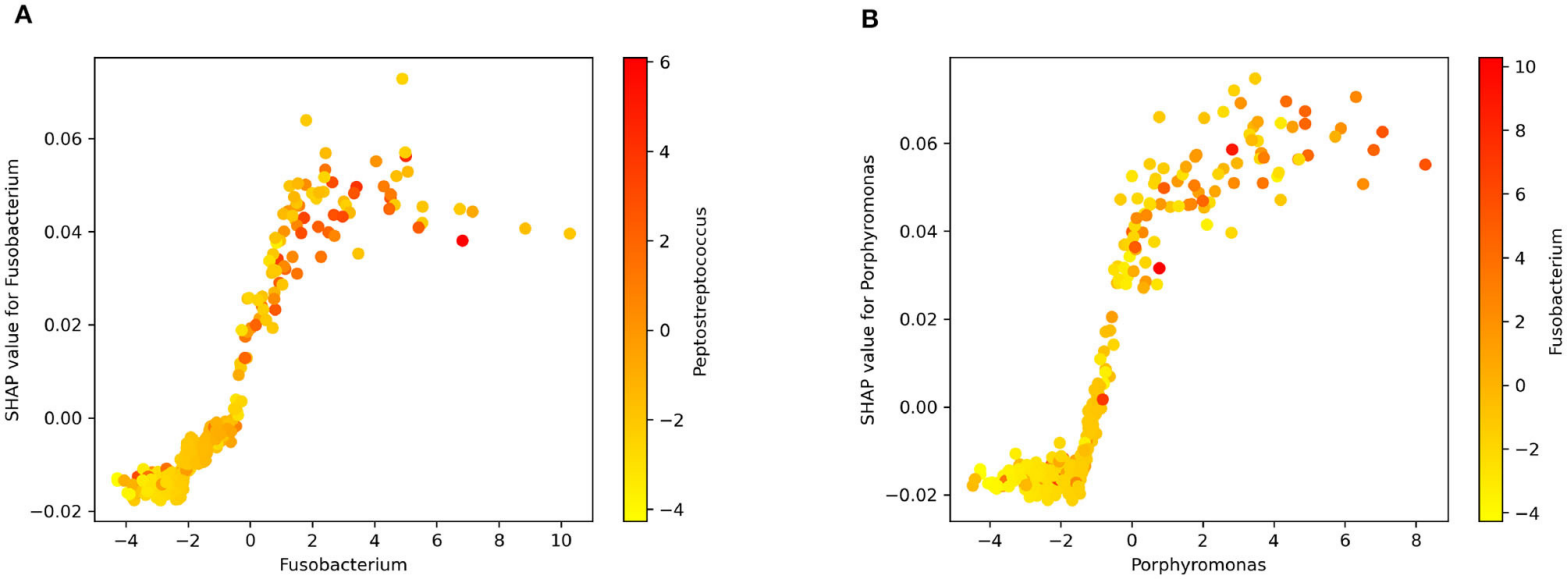
SHAP dependence plot for **(A)** *Fusobacterium* and *Peptostreptococcus*. **(B)** *Porphyromonas* and *Fusobacterium*.

Figure 5 displays the dependence plots for the top two variables according to the SHAP summary plot. Notably, the dependence of marginal contributions for a specific variable varies with the fluctuations in the variable itself. Specifically, in the depicted dependence plots, an increase in the values of *Fusobacterium* (Figure 5A) or *Porphyromonas* (Figure 5B) corresponds to a rise in the associated SHAP values. Consequently, elevated values of these variables play a significant role in the algorithm's decision to classify an instance as CRC. Moreover, the color code represents the abundance of another bacterium. In Figures 5A, B can be observed the correlation of *Fusobacterium* with *Peptostreptococcus* and *Porphyromonas*, respectively.

## 5 Discussion

In our research, we have crafted an Artificial Intelligence workflow adept at deciphering the human microbiome within a cohort of control and CRC subjects, offering a highly dependable prediction of CRC outcomes. A notable strength lies in the entirely data-driven implementation of the classifier. Additionally, the preprocessing pipeline impartially eliminates less informative bacteria without relying on diagnostic labels associated with the microbiome. Beyond its precision, the top classifier yields predictions that are readily interpretable. XAI analysis results reveal a discernible pattern aligning with established knowledge, highlighting some bacterial genera among the 20 most significant features, known for their association with CRC in existing literature.

Among the foremost 20 features, *Fusobacterium, Porphyromonas, Peptostreptococcus*, and *Parvimonas* have emerged as potential microbiological markers that could significantly improve the accuracy of colorectal cancer (CRC) diagnoses (Chen et al., 2022).

Figure 5 offers insight into the connection between specific bacterial genera and CRC. The observed positive correlation between the relative abundance of well-documented bacteria like *Fusobacterium* and *Porphyromonas* and SHAP values suggests their influence on the model's predictions. This correlation hints at the biological relevance of these taxa in the context of CRC. Essentially, a higher abundance of these bacteria appears to positively impact the model's attribution of the positive class (cancer) during output explanation. The visual representation in Figure 5 aids in understanding the model's decision-making from a biological standpoint (Zhou et al., 2018; Koliarakis et al., 2019).

The recognition of abundant bacteria originating from the oral cavity, including *Fusobacterium, Peptostreptococcus*, and *Parvimonas*, indicates a dynamic symbiotic metacommunity intricately linked to the initiation of colorectal cancer (CRC). Within the human body, a symbiotic relationship with the microbiota exists, where polymicrobial communities inhabit cavities such as the oral and intestinal regions. Despite these areas being anatomically separated with distinct microbiota colonization, there are indications that bacteria from the oral cavity may migrate to the colon (Koliarakis et al., 2019). *Fusobacterium* has been associated with genetic and epigenetic abnormalities in colorectal cancer (CRC) tissues, including microsatellite instability (MSI). In the tumorigenesis and progression of CRC, Fusobacterium has the potential to enhance proliferation and metabolism, alter the immune microenvironment, and promote metastasis and chemoresistance. It may serve as a biomarker for identifying individuals at high risk for CRC (Wang and Fang, 2023).

According to our study, a high concentration of bacteria from the *Lachnospiraceae* family is associated with a lower likelihood of CRC. This spurious association has been observed in previous works, including (Hexun et al., 2023; Zhang et al., 2023), and this could be linked to the mechanism whereby a high concentration of these bacteria may promote heightened immune surveillance, thus controlling colorectal cancer progression and counteracting it.

Additionally, from the summary plot, we observe another pattern well-documented in the literature. There are studies indicating that certain bacteria of the *Clostridiales* order, including *Eubacterium eligens, Eubacterium ventriosum*, and *Anaerostipes*, are significantly reduced in CRC patients compared to control subjects (Montalban-Arques et al., 2021). This is evident in Figure 4B, where corresponding to these commensal bacteria, the high concentration of these bacteria (red points on the plot) is associated with negative SHAP values, indicating that the model assigns a low probability of classifying these subjects as CRC.

Regarding demographic descriptors, age, gender, and BMI have emerged as important features. Higher age, male gender, and elevated BMI appear to be positively associated with CRC. These findings are widely accepted and supported by scientific literature, where obesity is recognized as a factor associated with the development of this tumor, along with advancing age. Age exhibits a consistent trend with expected associations: longer lifespans correspond to a higher risk of having CRC (Murphy et al., 2011; Ye et al., 2020; Elangovan et al., 2021).

In addition to the strengths mentioned above, we performed a comprehensive analysis of explainability across the three models employed in our study. This analysis, as can be observed in Figure 4B and in Supplementary Figure S5, demonstrates the comparability of explainability results in terms of both the most important features and the correlation between feature values and their corresponding Shap values. Notably, the positive/negative correlations observed between SHAP values and the abundance of specific features persist consistently across all three models.

This consistency in the interpretability of our models enhances the robustness of our findings.

The presented study acknowledges certain limitations that we aim to address in future research efforts. While the classification performance provides valuable insights, there is the potential for further optimization. This could be attributed to the presence of other factors associated with colorectal cancer, such as hereditary factors and smoking, which were not considered in our analysis. Furthermore, the utilized database, obtained through 16S rRNA sequencing, provides a limited taxonomic resolution compared to Shotgun sequencing. A finer taxonomic resolution might have contributed to a more precise analysis and potentially identified stronger associations with the disease.

In the realm of CRC research, our study takes a distinctive approach by applying XAI techniques to unravel the intricate relationship between the human microbiome and CRC. Utilizing SHAP in microbiome research for predicting CRC outcomes enhances the transparency of our model and introduces a new perspective for the application of XAI in personalized medicine. Our identification of microbiological markers and taxonomic units associated with CRC risk contributes to the understanding of disease mechanisms and has the potential to inform diagnostic and therapeutic strategies. By acknowledging demographic descriptors alongside microbiome features, our work ensures a comprehensive approach that can be applicable across diverse patient populations. In recognizing the challenges and limitations of our study, we aim to guide future investigations, emphasizing our commitment to advancing both the scientific understanding of CRC and the practical applications of contemporary technologies.

## 6 Conclusion

This study has enabled the identification of bacteria that significantly influence the discrimination between healthy and diseased individuals through Explainable Artificial Intelligence (XAI), suggesting the identification of new disease biomarkers.

Additionally, the use of explainable artificial intelligence models can support making these models more transparent and interpretable, allowing for the appreciation, understanding, and utilization of the microbiota composition for each individual. By employing such the proposed method for each subject, an assessment of the microbiota can be conducted, with the aim of implementing actions to evaluate its modification, if necessary.

## Data availability statement

Publicly available datasets were analyzed in this study. The datasets analyzed for this study can be found in the Zenodo repository (Marcos-Zambrano, 2022), and via https://github.com/pierfrancesco2021/XAI-for-Microbiome-Data-Analysis-in-CRC.

## Author contributions

PN: Conceptualization, Data curation, Formal analysis, Investigation, Methodology, Software, Visualization, Writing – original draft, Writing – review & editing. DR: Data curation, Methodology, Writing – original draft. MM: Data curation, Methodology, Writing – original draft. PB: Writing – original draft, Writing – review & editing. DD: Writing – review & editing. AC: Formal analysis, Methodology, Writing – review & editing. GL: Writing – review & editing. DS: Writing – review & editing. VV: Writing – review & editing. PF: Writing – review & editing. RB: Supervision, Writing – review & editing. MD: Supervision, Writing – review & editing. FI: Writing – review & editing. ST: Conceptualization, Funding acquisition, Investigation, Methodology, Project administration, Supervision, Visualization, Writing – original draft, Writing – review & editing.
