# Supplementary material for: Explainable artificial intelligence for microbiome data analysis in colorectal cancer biomarker identification
Source: Front Microbiol. 2024 Feb 15;15:1348974. doi: 10.3389/fmicb.2024.1348974 (PMC10901987; doi:10.3389/fmicb.2024.1348974)
Supplement: Supplementary file 1 [file Data_Sheet_1.PDF]

Supplementary Material

1 SUPPLEMENTARY TABLES AND FIGURES

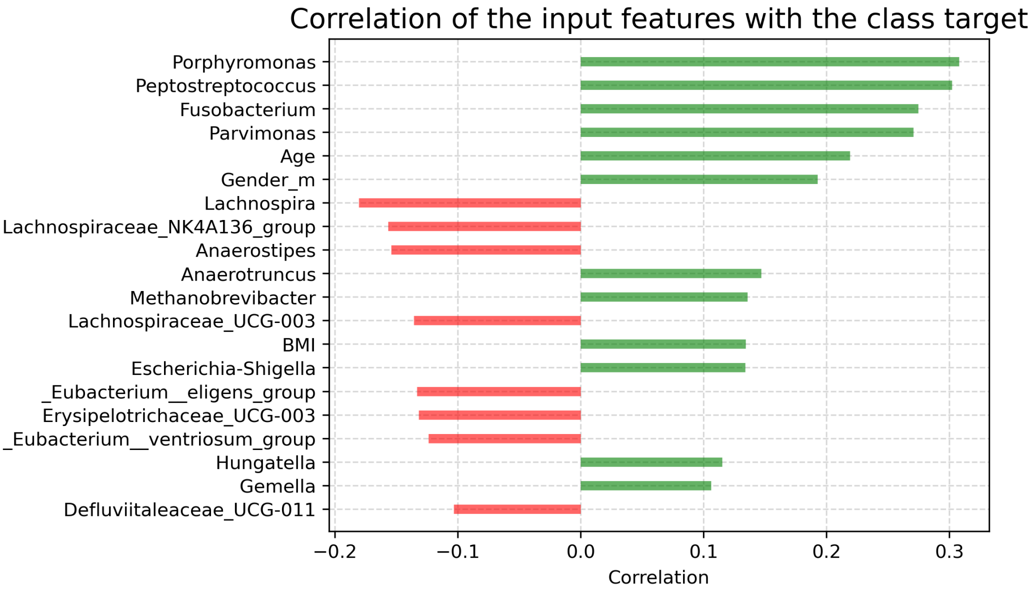

Figure S1: Representation of the top 20 features ranked according to the correlation coefficient between each variable and the target class.

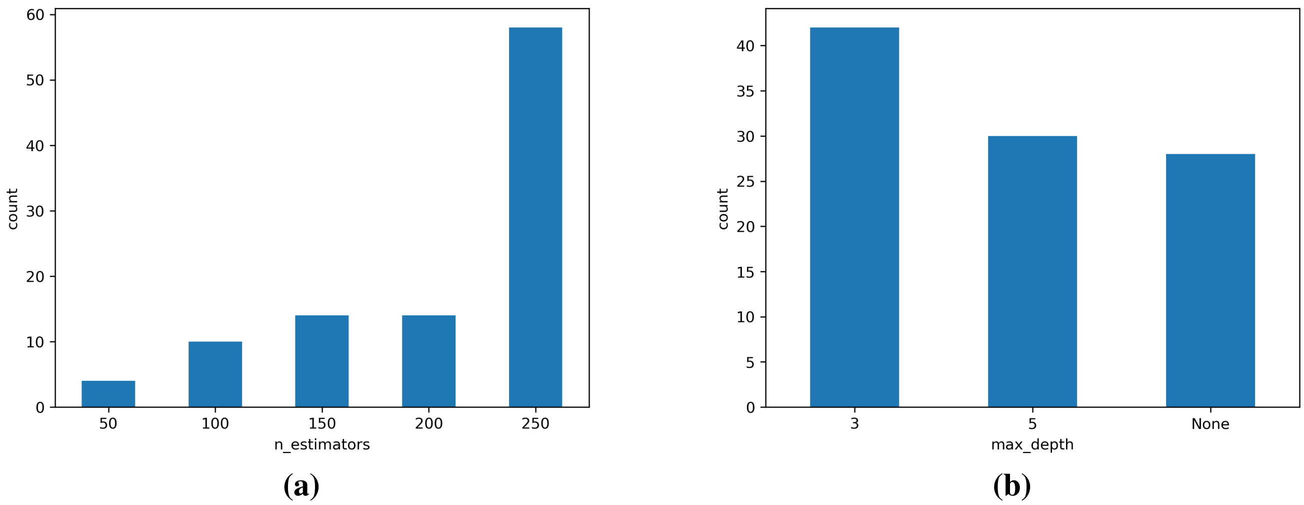

Figure S2: Stability analysis of tuned parameters in nested cross-validation for RF: **(a)** n_estimators **(b)** max_depth.

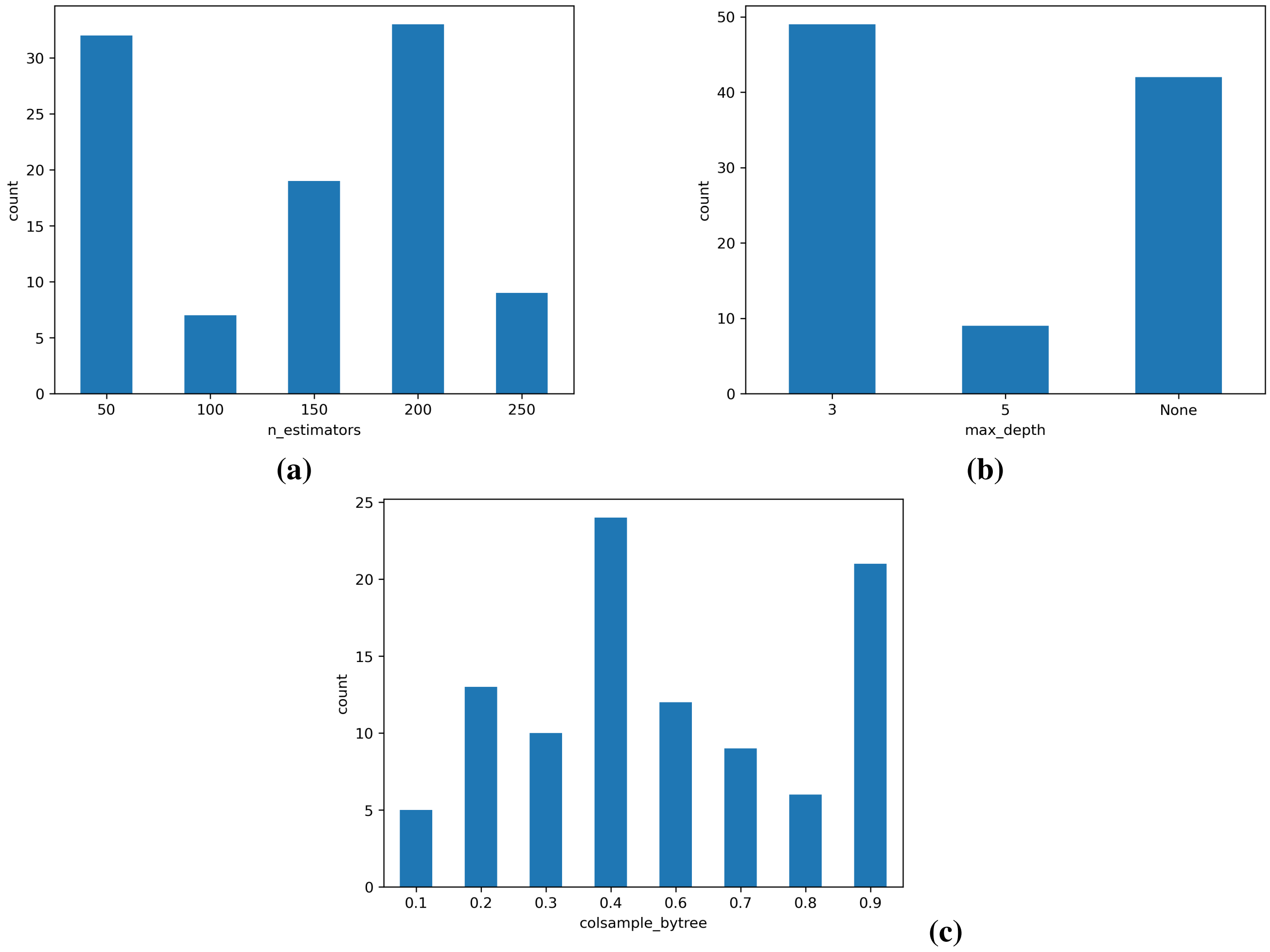

Figure S3: Stability analysis of tuned parameters in nested cross-validation for XGBoost: **(a)** n_estimators **(b)** max_depth **(c)** colsample_bytree.

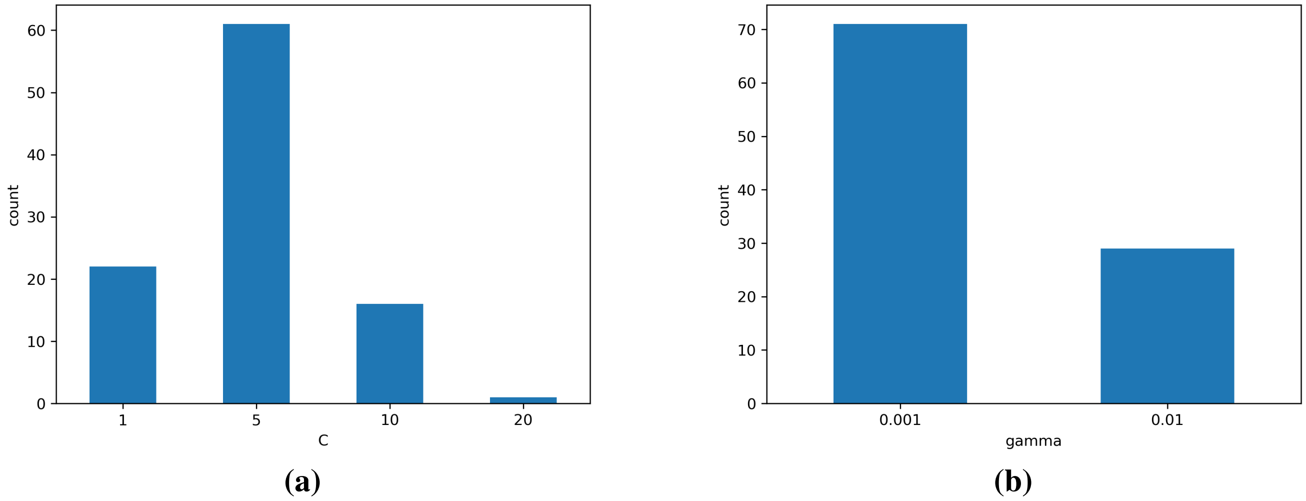

Figure S4: Stability analysis of tuned parameters in nested cross-validation for SVM: **(a)** C **(b)** gamma.

Table S1: Metadata information about subjects involved in the analysis.

SampleID	Diagnosis	Study	Country	Age	BMI	Gender_m
**Cancer1-2355**	Cancer	Zackular	USA	70	23.0	1
**Cancer10-2567**	Cancer	Zackular	USA	69	27.0	1
**Cancer11-2573**	Cancer	Zackular	USA	47	26.0	0
**Cancer12-2575**	Cancer	Zackular	USA	67	32.0	0
**Cancer13-2579**	Cancer	Zackular	USA	55	57.0	1
**Cancer14-2611**	Cancer	Zackular	USA	49		1
**Cancer15-2613**	Cancer	Zackular	USA	61	35.0	1
**Cancer16-2619**	Cancer	Zackular	USA	63	31.0	1
**Cancer17-2625**	Cancer	Zackular	USA	43	24.0	1
**Cancer18-2629**	Cancer	Zackular	USA	52	29.0	1
**Cancer19-2633**	Cancer	Zackular	USA	71	32.0	1
**Cancer2-2455**	Cancer	Zackular	USA	59	29.0	1
**Cancer20-2667**	Cancer	Zackular	USA	58	30.0	1
**Cancer21-2671**	Cancer	Zackular	USA	60	33.0	1
**Cancer22-2703**	Cancer	Zackular	USA	51	24.0	0
**Cancer23-2705**	Cancer	Zackular	USA	64	24.0	0
**Cancer24-2727**	Cancer	Zackular	USA	48	33.0	1
**Cancer25-2735**	Cancer	Zackular	USA	60	28.0	0
**Cancer26-2763**	Cancer	Zackular	USA	43	46.0	1
**Cancer27-2771**	Cancer	Zackular	USA	59	30.0	0
**Cancer28-2777**	Cancer	Zackular	CA	73	22.0	0
**Cancer29-2801**	Cancer	Zackular	USA	59	23.0	1
**Cancer3-2513**	Cancer	Zackular	USA	69	28.0	1
**Cancer30-2863**	Cancer	Zackular	CA	57	34.0	1
**Cancer4-2521**	Cancer	Zackular	USA	88	26.0	1
**Cancer5-2523**	Cancer	Zackular	USA	37	35.0	1
**Cancer6-2535**	Cancer	Zackular	USA	69	26.0	1
**Cancer7-2537**	Cancer	Zackular	USA	74	32.0	0
**Cancer8-2543**	Cancer	Zackular	USA	56	33.0	1
**Cancer9-2547**	Cancer	Zackular	USA	51	35.0	0
**ERR475468**	Normal	Zeller	FRA	74	27.0	1
**ERR475469**	Normal	Zeller	FRA	54	26.0	1
**ERR475470**	Normal	Zeller	FRA	65	30.0	1
**ERR475471**	Normal	Zeller	FRA	57	24.0	1
**ERR475473**	Normal	Zeller	FRA	53	30.0	0
**ERR475474**	Normal	Zeller	FRA	62	21.0	0
**ERR475476**	Normal	Zeller	FRA	53	32.0	1
**ERR475477**	Normal	Zeller	FRA	68	26.0	1
**ERR475478**	Normal	Zeller	FRA	59	25.0	0
**ERR475480**	Normal	Zeller	FRA	72	24.0	1
**ERR475481**	Normal	Zeller	FRA	55	20.0	0
**ERR475482**	Cancer	Zeller	FRA	72	37.0	0

**ERR475483**	Normal	Zeller	FRA	63	27.0	0
**ERR475484**	Normal	Zeller	FRA	69	25.0	0
**ERR475485**	Normal	Zeller	FRA	61	24.0	0
**ERR475486**	Normal	Zeller	FRA	68	25.0	0
**ERR475487**	Normal	Zeller	FRA	63	21.0	0
**ERR475488**	Normal	Zeller	FRA	62	24.0	1
**ERR475491**	Normal	Zeller	FRA	64	27.0	1
**ERR475492**	Normal	Zeller	FRA	65	24.0	0
**ERR475493**	Cancer	Zeller	FRA	62	21.0	0
**ERR475494**	Normal	Zeller	FRA	59	25.0	0
**ERR475495**	Normal	Zeller	FRA	70	25.0	1
**ERR475497**	Normal	Zeller	FRA	66	28.0	0
**ERR475499**	Normal	Zeller	FRA	63	25.0	1
**ERR475500**	Cancer	Zeller	FRA	58	24.0	1
**ERR475501**	Cancer	Zeller	FRA	63	22.0	1
**ERR475503**	Normal	Zeller	FRA	67	27.0	1
**ERR475504**	Normal	Zeller	FRA	61	23.0	0
**ERR475508**	Normal	Zeller	FRA	71	23.0	0
**ERR475509**	Cancer	Zeller	FRA	79	30.0	1
**ERR475512**	Normal	Zeller	FRA	63		1
**ERR475513**	Cancer	Zeller	FRA	50	24.0	0
**ERR475514**	Normal	Zeller	FRA	84	23.0	1
**ERR475515**	Cancer	Zeller	FRA	73	24.0	0
**ERR475516**	Cancer	Zeller	FRA	59	27.0	1
**ERR475517**	Normal	Zeller	FRA	50	25.0	1
**ERR475519**	Normal	Zeller	FRA	62	23.0	1
**ERR475520**	Cancer	Zeller	FRA	73	26.0	0
**ERR475521**	Cancer	Zeller	FRA	48	24.0	0
**ERR475522**	Normal	Zeller	FRA	61	23.0	1
**ERR475523**	Normal	Zeller	FRA	61	34.0	0
**ERR475524**	Normal	Zeller	FRA	67	23.0	0
**ERR475525**	Normal	Zeller	FRA	63	22.0	0
**ERR475526**	Normal	Zeller	FRA	67	21.0	1
**ERR475527**	Cancer	Zeller	FRA	63	25.0	1
**ERR475529**	Cancer	Zeller	FRA	80	29.0	1
**ERR475531**	Cancer	Zeller	FRA	44	20.0	1
**ERR475532**	Cancer	Zeller	FRA	78	19.0	1
**ERR475533**	Cancer	Zeller	FRA	63	29.0	1
**ERR475534**	Normal	Zeller	FRA	77	22.0	0
**ERR475539**	Cancer	Zeller	FRA	87	15.0	0
**ERR475540**	Cancer	Zeller	FRA	73	17.0	1
**ERR475541**	Cancer	Zeller	FRA	60	26.0	1
**ERR475545**	Normal	Zeller	FRA	46	29.0	0

**ERR475547**	Cancer	Zeller	FRA	72	22.0	1
**ERR475548**	Cancer	Zeller	FRA	55	25.0	0
**ERR475549**	Normal	Zeller	FRA	67	28.0	0
**ERR475550**	Normal	Zeller	FRA	35	23.0	1
**ERR475553**	Cancer	Zeller	FRA	80	23.0	0
**ERR475555**	Cancer	Zeller	FRA	51	24.0	1
**ERR475557**	Cancer	Zeller	FRA	51	30.0	1
**ERR475560**	Cancer	Zeller	FRA	67	25.0	1
**ERR475562**	Normal	Zeller	FRA	65	30.0	1
**ERR475563**	Normal	Zeller	FRA	67	25.0	1
**ERR475564**	Normal	Zeller	FRA	52	24.0	1
**ERR475565**	Normal	Zeller	FRA	60	28.0	0
**ERR475566**	Normal	Zeller	FRA	49	23.0	0
**ERR475567**	Normal	Zeller	FRA	62	20.0	0
**ERR475568**	Cancer	Zeller	FRA	45	28.0	0
**ERR475569**	Cancer	Zeller	FRA	79	22.0	0
**ERR475570**	Normal	Zeller	FRA	72	25.0	0
**ERR475571**	Normal	Zeller	FRA	64	30.0	0
**ERR475572**	Normal	Zeller	FRA	38	22.0	0
**ERR475573**	Cancer	Zeller	FRA	64	30.0	1
**ERR475574**	Normal	Zeller	FRA	66	25.0	1
**ERR475575**	Normal	Zeller	FRA	74	27.0	1
**ERR475576**	Cancer	Zeller	FRA	69	24.0	0
**ERR475577**	Cancer	Zeller	FRA	69	30.0	0
**ERR475578**	Cancer	Zeller	FRA	75	37.0	1
**ERR475579**	Cancer	Zeller	FRA	69	25.0	0
**ERR475580**	Cancer	Zeller	FRA	68	23.0	1
**ERR475581**	Cancer	Zeller	FRA	65	26.0	1
**ERR475582**	Cancer	Zeller	FRA	62	26.0	1
**ERR475585**	Cancer	Zeller	FRA	73	40.0	1
**ERR475586**	Cancer	Zeller	FRA	74	19.0	0
**ERR475588**	Cancer	Zeller	FRA	74	26.0	0
**ERR475589**	Cancer	Zeller	FRA	65	24.0	1
**ERR475591**	Cancer	Zeller	FRA	53	25.0	1
**ERR475592**	Cancer	Zeller	FRA	67	28.0	1
**ERR475594**	Cancer	Zeller	FRA	54	22.0	0
**Healthy1-2027**	Normal	Zackular	CA	65	22.0	0
**Healthy10-3087**	Normal	Zackular	USA	51	25.0	1
**Healthy11-3123**	Normal	Zackular	USA	55	38.0	0
**Healthy12-3211**	Normal	Zackular	USA	51	30.0	0
**Healthy13-3241**	Normal	Zackular	USA	45	33.0	0
**Healthy14-3245**	Normal	Zackular	USA	61	21.0	0
**Healthy15-3247**	Normal	Zackular	USA	51	21.0	0

**Healthy16-3251**	Normal	Zackular	USA	57	24.0	0
**Healthy17-3257**	Normal	Zackular	USA	65	33.0	1
**Healthy18-3293**	Normal	Zackular	USA	52	32.0	0
**Healthy19-3339**	Normal	Zackular	USA	54	34.0	0
**Healthy2-2779**	Normal	Zackular	CA	69	19.0	0
**Healthy20-3341**	Normal	Zackular	USA	75	24.0	0
**Healthy21-3347**	Normal	Zackular	USA	61	25.0	1
**Healthy22-3363**	Normal	Zackular	USA	58	29.0	1
**Healthy23-3367**	Normal	Zackular	USA	56	21.0	0
**Healthy24-3403**	Normal	Zackular	USA	51	21.0	1
**Healthy25-3405**	Normal	Zackular	USA	42	28.0	1
**Healthy26-3407**	Normal	Zackular	USA	49	25.0	1
**Healthy27-3439**	Normal	Zackular	USA	53	22.0	0
**Healthy28-3499**	Normal	Zackular	USA	46	21.0	0
**Healthy29-3529**	Normal	Zackular	USA	51	30.0	0
**Healthy3-3021**	Normal	Zackular	USA	54	32.0	1
**Healthy30-3291**	Normal	Zackular	USA	63	33.0	0
**Healthy4-3023**	Normal	Zackular	USA	44	26.0	0
**Healthy5-3043**	Normal	Zackular	USA	52	21.0	0
**Healthy6-3061**	Normal	Zackular	USA	75	25.0	1
**Healthy7-3063**	Normal	Zackular	USA	52	25.0	1
**Healthy8-3073**	Normal	Zackular	CA	65	27.0	1
**Healthy9-3075**	Normal	Zackular	USA	35	35.0	0
**SRR2143516**	Normal	Baxter	USA	64	36.0	1
**SRR2143517**	Normal	Baxter	USA	61	28.0	1
**SRR2143518**	Normal	Baxter	USA	47	22.0	0
**SRR2143521**	Normal	Baxter	USA	44	25.0	0
**SRR2143522**	Normal	Baxter	USA	51	26.0	0
**SRR2143523**	Cancer	Baxter	USA	78	26.0	1
**SRR2143524**	Normal	Baxter	USA	59	21.0	1
**SRR2143525**	Normal	Baxter	USA	63	23.0	0
**SRR2143526**	Cancer	Baxter	USA	67	21.0	1
**SRR2143527**	Normal	Baxter	USA	65	22.0	0
**SRR2143531**	Normal	Baxter	USA	77	22.0	1
**SRR2143533**	Cancer	Baxter	USA	88	23.0	0
**SRR2143534**	Normal	Baxter	USA	56	32.0	0
**SRR2143536**	Normal	Baxter	USA	63	23.0	1
**SRR2143537**	Normal	Baxter	USA	59	26.0	0
**SRR2143541**	Normal	Baxter	USA	59	26.0	1
**SRR2143544**	Normal	Baxter	USA	40	47.0	0
**SRR2143545**	Normal	Baxter	USA	67	32.0	0
**SRR2143548**	Normal	Baxter	USA	52	18.0	0
**SRR2143549**	Normal	Baxter	USA	49	26.0	0

**SRR2143552**	Normal	Baxter	USA	39	23.0	0
**SRR2143553**	Normal	Baxter	USA	43	24.0	0
**SRR2143554**	Normal	Baxter	USA	29	18.0	0
**SRR2143555**	Normal	Baxter	USA	32	35.0	0
**SRR2143556**	Normal	Baxter	USA	35	33.0	0
**SRR2143557**	Normal	Baxter	USA	42	27.0	1
**SRR2143558**	Normal	Baxter	USA	51	24.0	1
**SRR2143559**	Normal	Baxter	USA	59	27.0	0
**SRR2143560**	Normal	Baxter	USA	49	27.0	1
**SRR2143561**	Normal	Baxter	USA	77	32.0	0
**SRR2143562**	Normal	Baxter	USA	48	41.0	0
**SRR2143564**	Normal	Baxter	USA	37	34.0	0
**SRR2143566**	Normal	Baxter	USA	62	32.0	0
**SRR2143570**	Normal	Baxter	USA	57	24.0	1
**SRR2143574**	Normal	Baxter	USA	52	33.0	0
**SRR2143577**	Normal	Baxter	USA	62	27.0	1
**SRR2143578**	Normal	Baxter	USA	55	36.0	0
**SRR2143579**	Normal	Baxter	USA	44	22.0	0
**SRR2143581**	Normal	Baxter	USA	48	27.0	1
**SRR2143584**	Normal	Baxter	USA	54	25.0	1
**SRR2143586**	Normal	Baxter	USA	55	18.0	0
**SRR2143587**	Normal	Baxter	USA	51	21.0	0
**SRR2143588**	Normal	Baxter	USA	55	20.0	0
**SRR2143589**	Normal	Baxter	USA	61	24.0	1
**SRR2143590**	Normal	Baxter	USA	56	27.0	1
**SRR2143591**	Normal	Baxter	USA	36	34.0	0
**SRR2143593**	Normal	Baxter	USA	56	26.0	0
**SRR2143596**	Normal	Baxter	USA	45	29.0	1
**SRR2143599**	Cancer	Baxter	USA	78	25.0	0
**SRR2143600**	Normal	Baxter	USA	76	37.0	0
**SRR2143601**	Normal	Baxter	USA	72	22.0	1
**SRR2143602**	Normal	Baxter	USA	70	31.0	1
**SRR2143605**	Normal	Baxter	USA	49	25.0	0
**SRR2143606**	Cancer	Baxter	USA	45	30.0	1
**SRR2143609**	Normal	Baxter	USA	35	24.0	1
**SRR2143613**	Normal	Baxter	USA	63	18.0	0
**SRR2143621**	Cancer	Baxter	USA	72	24.0	1
**SRR2143622**	Normal	Baxter	USA	64	29.0	1
**SRR2143627**	Cancer	Baxter	USA	54	27.0	1
**SRR2143630**	Normal	Baxter	USA	47	26.0	0
**SRR2143631**	Normal	Baxter	USA	62	40.0	1
**SRR2143633**	Cancer	Baxter	USA	60	23.0	0
**SRR2143634**	Cancer	Baxter	USA	71	28.0	0

**SRR2143635**	Cancer	Baxter	USA	64	23.0	1
**SRR2143636**	Normal	Baxter	USA	48	38.0	0
**SRR2143637**	Cancer	Baxter	USA	75	28.0	0
**SRR2143638**	Normal	Baxter	USA	35	24.0	0
**SRR2143644**	Cancer	Baxter	USA	71	27.0	1
**SRR2143647**	Normal	Baxter	USA	42	23.0	0
**SRR2143649**	Normal	Baxter	USA	37	21.0	0
**SRR2143650**	Normal	Baxter	USA	41	20.0	1
**SRR2143652**	Cancer	Baxter	USA	80	28.0	0
**SRR2143654**	Normal	Baxter	USA	55	27.0	0
**SRR2143657**	Normal	Baxter	USA	62	21.0	0
**SRR2143659**	Cancer	Baxter	USA	45	24.0	1
**SRR2143660**	Normal	Baxter	USA	59	23.0	0
**SRR2143662**	Cancer	Baxter	USA	72	31.0	1
**SRR2143663**	Cancer	Baxter	USA	78	27.0	0
**SRR2143664**	Normal	Baxter	USA	70	31.0	1
**SRR2143665**	Cancer	Baxter	USA	70	23.0	1
**SRR2143667**	Cancer	Baxter	USA	62	30.0	0
**SRR2143668**	Normal	Baxter	USA	64	31.0	1
**SRR2143669**	Cancer	Baxter	USA	80	30.0	0
**SRR2143672**	Normal	Baxter	USA	60	32.0	1
**SRR2143674**	Cancer	Baxter	USA	85	28.0	1
**SRR2143675**	Cancer	Baxter	USA	78	25.0	1
**SRR2143676**	Cancer	Baxter	USA	84	32.0	0
**SRR2143677**	Cancer	Baxter	USA	80	27.0	1
**SRR2143681**	Cancer	Baxter	USA	68	27.0	1
**SRR2143682**	Cancer	Baxter	USA	47	29.0	0
**SRR2143683**	Normal	Baxter	USA	56	23.0	0
**SRR2143684**	Cancer	Baxter	USA	62	26.0	1
**SRR2143693**	Cancer	Baxter	USA	59	29.0	1
**SRR2143694**	Cancer	Baxter	USA	59	30.0	1
**SRR2143696**	Cancer	Baxter	USA	46	26.0	1
**SRR2143697**	Cancer	Baxter	USA	32	33.0	1
**SRR2143698**	Cancer	Baxter	USA	61	21.0	0
**SRR2143699**	Normal	Baxter	USA	46	28.0	0
**SRR2143705**	Cancer	Baxter	USA	85	26.0	1
**SRR2143707**	Cancer	Baxter	USA	57	27.0	1
**SRR2143709**	Cancer	Baxter	USA	60	56.0	1
**SRR2143712**	Cancer	Baxter	USA	69	28.0	1
**SRR2143716**	Cancer	Baxter	USA	37	35.0	1
**SRR2143717**	Cancer	Baxter	USA	79	26.0	0
**SRR2143718**	Cancer	Baxter	USA	53	30.0	1
**SRR2143719**	Cancer	Baxter	USA	75	30.0	0

**SRR2143721**	Cancer	Baxter	USA	69	26.0	1
**SRR2143722**	Cancer	Baxter	USA	74	32.0	0
**SRR2143723**	Cancer	Baxter	USA	52	34.0	1
**SRR2143724**	Cancer	Baxter	USA	64	24.0	0
**SRR2143725**	Cancer	Baxter	USA	56	33.0	1
**SRR2143727**	Cancer	Baxter	USA	51	35.0	0
**SRR2143732**	Cancer	Baxter	USA	46	20.0	0
**SRR2143734**	Cancer	Baxter	USA	64	16.0	0
**SRR2143735**	Cancer	Baxter	USA	67	32.0	0
**SRR2143736**	Cancer	Baxter	USA	55	57.0	1
**SRR2143743**	Normal	Baxter	USA	55	28.0	0
**SRR2143744**	Cancer	Baxter	USA	49		1
**SRR2143745**	Cancer	Baxter	USA	61	35.0	1
**SRR2143746**	Cancer	Baxter	USA	60	29.0	1
**SRR2143747**	Cancer	Baxter	USA	63	31.0	1
**SRR2143748**	Cancer	Baxter	USA	43	24.0	1
**SRR2143749**	Cancer	Baxter	USA	52	29.0	1
**SRR2143750**	Cancer	Baxter	USA	71	32.0	1
**SRR2143758**	Normal	Baxter	USA	51	28.0	1
**SRR2143759**	Cancer	Baxter	USA	58	30.0	1
**SRR2143760**	Normal	Baxter	USA	51	36.0	0
**SRR2143761**	Cancer	Baxter	USA	60	33.0	1
**SRR2143762**	Normal	Baxter	USA	62	31.0	1
**SRR2143764**	Normal	Baxter	USA	44	28.0	0
**SRR2143766**	Normal	Baxter	USA	51	19.0	0
**SRR2143767**	Normal	Baxter	USA	50	33.0	0
**SRR2143768**	Cancer	Baxter	USA	51	21.0	0
**SRR2143769**	Cancer	Baxter	USA	64	24.0	0
**SRR2143770**	Cancer	Baxter	USA	65	27.0	0
**SRR2143776**	Cancer	Baxter	USA	48	33.0	1
**SRR2143777**	Normal	Baxter	USA	52	28.0	0
**SRR2143778**	Normal	Baxter	USA	41	22.0	1
**SRR2143779**	Cancer	Baxter	USA	65	32.0	1
**SRR2143780**	Normal	Baxter	USA	44	25.0	0
**SRR2143784**	Cancer	Baxter	USA	78	31.0	1
**SRR2143785**	Cancer	Baxter	USA	59	30.0	0
**SRR2143786**	Cancer	Baxter	USA	63	29.0	1
**SRR2143788**	Cancer	Baxter	USA	73	22.0	0
**SRR2143789**	Normal	Baxter	USA	69	19.0	0
**SRR2143792**	Cancer	Baxter	USA	64	35.0	0
**SRR2143796**	Cancer	Baxter	USA	59	23.0	1
**SRR2143798**	Cancer	Baxter	USA	68	30.0	0
**SRR2143805**	Cancer	Baxter	USA	59	50.0	0

**SRR2143807**	Cancer	Baxter	USA	36	21.0	0
**SRR2143811**	Normal	Baxter	USA	31	24.0	1
**SRR2143813**	Cancer	Baxter	USA	58	22.0	1
**SRR2143814**	Cancer	Baxter	USA	65	43.0	0
**SRR2143818**	Normal	Baxter	USA	53	26.0	0
**SRR2143824**	Cancer	Baxter	USA	57	34.0	1
**SRR2143827**	Cancer	Baxter	USA	69	22.0	0
**SRR2143830**	Cancer	Baxter	USA	69	28.0	1
**SRR2143832**	Cancer	Baxter	USA	74	35.0	1
**SRR2143835**	Normal	Baxter	USA	74	38.0	0
**SRR2143836**	Cancer	Baxter	USA	71	32.0	1
**SRR2143837**	Normal	Baxter	USA	52	35.0	0
**SRR2143838**	Normal	Baxter	USA	55	21.0	0
**SRR2143839**	Normal	Baxter	USA	56	29.0	1
**SRR2143840**	Cancer	Baxter	USA	81	23.0	0
**SRR2143841**	Normal	Baxter	USA	69	26.0	0
**SRR2143843**	Normal	Baxter	USA	58	27.0	0
**SRR2143854**	Cancer	Baxter	USA	40	25.0	0
**SRR2143855**	Cancer	Baxter	USA	75	26.0	1
**SRR2143856**	Cancer	Baxter	USA	50	28.0	1
**SRR2143863**	Normal	Baxter	USA	61	21.0	0
**SRR2143864**	Normal	Baxter	USA	50	27.0	0
**SRR2143881**	Normal	Baxter	USA	47	24.0	0
**SRR2143883**	Cancer	Baxter	USA	50	29.0	1
**SRR2143884**	Cancer	Baxter	USA	40	30.0	1
**SRR2143885**	Normal	Baxter	USA	40	34.0	1
**SRR2143886**	Cancer	Baxter	USA	51	26.0	1
**SRR2143888**	Normal	Baxter	USA	46	29.0	0
**SRR2143891**	Normal	Baxter	USA	36	22.0	0
**SRR2143892**	Normal	Baxter	USA	54	32.0	1
**SRR2143893**	Normal	Baxter	USA	44	26.0	0
**SRR2143895**	Cancer	Baxter	USA	86	28.0	0
**SRR2143896**	Normal	Baxter	USA	75	49.0	0
**SRR2143897**	Normal	Baxter	USA	52	21.0	0
**SRR2143898**	Normal	Baxter	USA	49	20.0	0
**SRR2143899**	Normal	Baxter	USA	51	28.0	0
**SRR2143900**	Normal	Baxter	USA	59	26.0	0
**SRR2143901**	Normal	Baxter	USA	50	34.0	0
**SRR2143903**	Normal	Baxter	USA	75	25.0	1
**SRR2143904**	Cancer	Baxter	USA	52	25.0	1
**SRR2143906**	Normal	Baxter	USA	60	35.0	1
**SRR2143907**	Normal	Baxter	USA	72	20.0	0
**SRR2143908**	Normal	Baxter	USA	55	26.0	0

**SRR2143909**	Cancer	Baxter	USA	35	35.0	0
**SRR2143910**	Normal	Baxter	USA	53	28.0	1
**SRR2143911**	Normal	Baxter	USA	57	21.0	0
**SRR2143912**	Normal	Baxter	USA	51	25.0	1
**SRR2143922**	Normal	Baxter	USA	62	25.0	0
**SRR2143924**	Cancer	Baxter	USA	57	33.0	1
**SRR2143925**	Normal	Baxter	USA	85	25.0	0
**SRR2143926**	Normal	Baxter	USA	45	21.0	0
**SRR2143929**	Normal	Baxter	USA	72	28.0	0
**SRR2143930**	Normal	Baxter	USA	72	23.0	0
**SRR2143932**	Cancer	Baxter	USA	79	21.0	1
**SRR2143933**	Cancer	Baxter	USA	88	31.0	0
**SRR2143934**	Normal	Baxter	USA	48	33.0	1
**SRR2143935**	Normal	Baxter	USA	55	38.0	0
**SRR2143940**	Cancer	Baxter	USA	68	32.0	0
**SRR2143941**	Normal	Baxter	USA	74	24.0	0
**SRR2143948**	Cancer	Baxter	USA	64	30.0	1
**SRR2143949**	Normal	Baxter	USA	54	29.0	1
**SRR2143955**	Normal	Baxter	USA	51	24.0	0
**SRR2143957**	Normal	Baxter	USA	80	22.0	1
**SRR2143961**	Normal	Baxter	USA	51	36.0	0
**SRR2143962**	Cancer	Baxter	USA	36	22.0	0
**SRR2143963**	Cancer	Baxter	USA	83	28.0	0
**SRR2143964**	Normal	Baxter	USA	76	31.0	1
**SRR2143965**	Normal	Baxter	USA	61	36.0	0
**SRR2143966**	Cancer	Baxter	USA	51	30.0	0
**SRR2143968**	Normal	Baxter	USA	67	26.0	0
**SRR2143969**	Normal	Baxter	USA	55	22.0	0
**SRR2143975**	Normal	Baxter	USA	71	28.0	1
**SRR2143977**	Normal	Baxter	USA	52	35.0	1
**SRR2143979**	Normal	Baxter	USA	52	18.0	0
**SRR2143980**	Normal	Baxter	USA	47	21.0	0
**SRR2143982**	Normal	Baxter	USA	62	27.0	1
**SRR2143983**	Normal	Baxter	USA	57	24.0	0
**SRR2143984**	Cancer	Baxter	USA	65	33.0	1
**SRR2143985**	Cancer	Baxter	USA	78	30.0	1
**SRR2143986**	Normal	Baxter	USA	61	24.0	1
**SRR2143992**	Cancer	Baxter	USA	67	23.0	1
**SRR2143996**	Cancer	Baxter	USA	52	20.0	0
**SRR2143997**	Normal	Baxter	USA	79	24.0	0
**SRR2143998**	Normal	Baxter	USA	63	33.0	0
**SRR2143999**	Cancer	Baxter	USA	52	32.0	0
**SRR2144001**	Cancer	Baxter	USA	71	27.0	0

**SRR2144003**	Normal	Baxter	USA	55	23.0	0
**SRR2144004**	Cancer	Baxter	USA	50	28.0	1
**SRR2144005**	Cancer	Baxter	USA	73	25.0	0
**SRR2144006**	Cancer	Baxter	USA	68	24.0	0
**SRR2144009**	Cancer	Baxter	USA	31	24.0	0
**SRR2144010**	Normal	Baxter	USA	84	21.0	0
**SRR2144011**	Normal	Baxter	USA	53	28.0	0
**SRR2144012**	Normal	Baxter	USA	54	26.0	0
**SRR2144013**	Cancer	Baxter	USA	53	25.0	0
**SRR2144014**	Normal	Baxter	USA	58	32.0	1
**SRR2144015**	Normal	Baxter	USA	54	34.0	0
**SRR2144017**	Normal	Baxter	USA	51	27.0	0
**SRR2144018**	Normal	Baxter	USA	55	30.0	0
**SRR2144019**	Normal	Baxter	USA	61	25.0	1
**SRR2144022**	Normal	Baxter	USA	63	29.0	1
**SRR2144024**	Normal	Baxter	USA	52	30.0	1
**SRR2144027**	Cancer	Baxter	USA	66	23.0	0
**SRR2144029**	Normal	Baxter	USA	61	27.0	0
**SRR2144030**	Cancer	Baxter	USA	61	28.0	1
**SRR2144031**	Cancer	Baxter	USA	69	36.0	1
**SRR2144032**	Cancer	Baxter	USA	45	30.0	0
**SRR2144033**	Cancer	Baxter	USA	88	27.0	1
**SRR2144037**	Normal	Baxter	USA	56	26.0	0
**SRR2144038**	Normal	Baxter	USA	51	21.0	1
**SRR2144039**	Normal	Baxter	USA	42	28.0	1
**SRR2144043**	Cancer	Baxter	USA	49	25.0	1
**SRR2144071**	Normal	Baxter	USA	77	24.0	1
**SRR2144074**	Normal	Baxter	USA	69	26.0	1
**SRR2144089**	Normal	Baxter	USA	51	18.0	0
**SRR2144090**	Normal	Baxter	USA	60	30.0	1
**SRR2144092**	Cancer	Baxter	USA	32	31.0	1
**SRR2144093**	Normal	Baxter	USA	50	28.0	1
**SRR2144094**	Cancer	Baxter	USA	74	27.0	1
**SRR2144096**	Normal	Baxter	USA	63	38.0	1
**SRR2144099**	Cancer	Baxter	USA	51	33.0	0
**SRR2144101**	Cancer	Baxter	USA	48	30.0	1
**SRR2144103**	Normal	Baxter	USA	44	36.0	1
**SRR2144104**	Normal	Baxter	USA	56	25.0	0
**SRR2144105**	Normal	Baxter	USA	55	33.0	1
**SRR2144106**	Normal	Baxter	USA	61	21.0	0
**SRR2144107**	Normal	Baxter	USA	67	29.0	1
**SRR2144108**	Cancer	Baxter	USA	62	27.0	1
**SRR2144109**	Normal	Baxter	USA	49	31.0	0

**SRR2144110**	Normal	Baxter	USA	52	23.0	0
**SRR2144112**	Normal	Baxter	USA	57	31.0	1
**SRR2144113**	Normal	Baxter	USA	46	21.0	0
**SRR2144115**	Cancer	Baxter	USA	50	31.0	0
**SRR2144117**	Normal	Baxter	USA	74	24.0	1
**SRR2144118**	Normal	Baxter	USA	53	21.0	0
**SRR2144119**	Normal	Baxter	USA	53	27.0	1
**SRR2144120**	Normal	Baxter	USA	56	21.0	1
**SRR2144123**	Normal	Baxter	USA	66	22.0	1
**SRR2144124**	Normal	Baxter	USA	77	32.0	1
**SRR2144125**	Normal	Baxter	USA	51	27.0	0
**SRR2144127**	Normal	Baxter	USA	75	27.0	1
**SRR2144129**	Normal	Baxter	USA	77	24.0	1

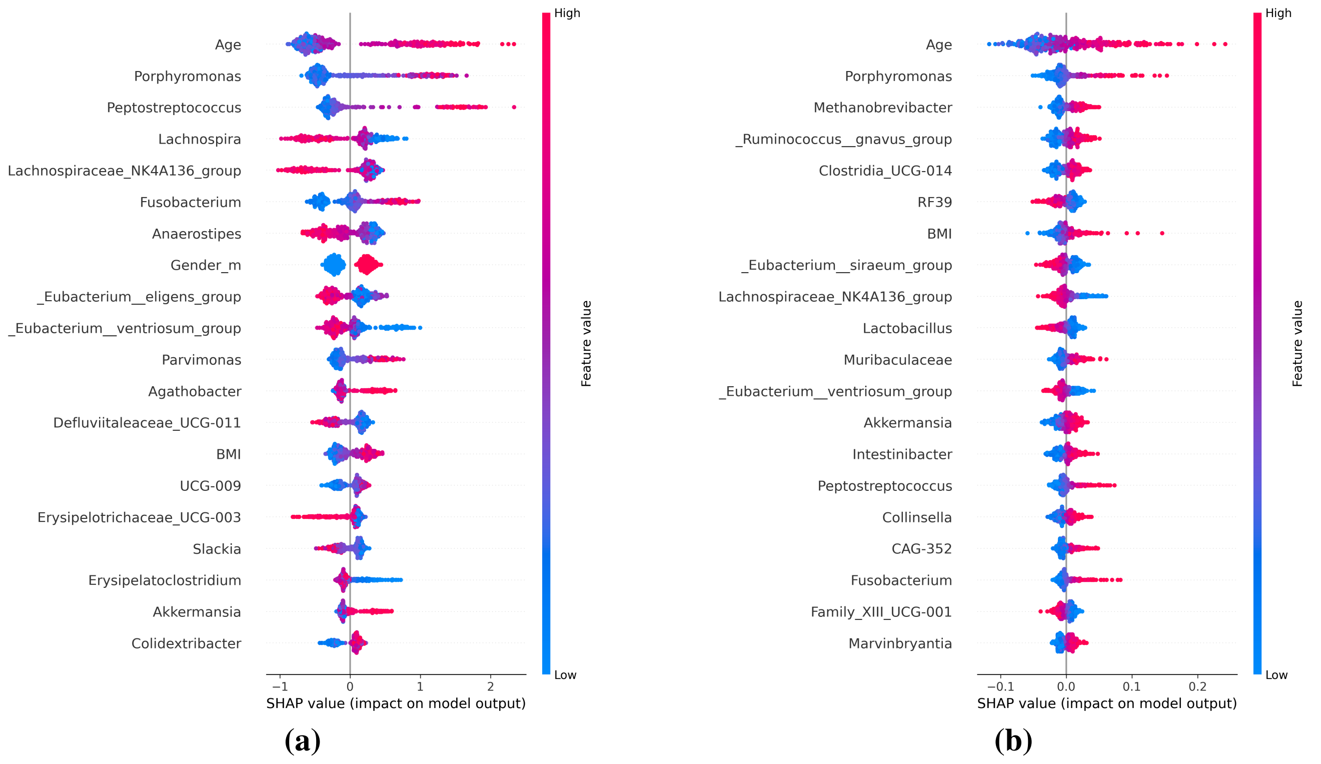

Figure S5: SHAP summary plot for: **(a)** XGBoost and **(b)** SVM. *The SHAP values for SVM were computed by repeating the cross-validation 5 times instead of 20 for computational reasons.
